# Adherence to the antirheumatic drugs: a systematic review and meta-analysis

**DOI:** 10.3389/fmed.2024.1456251

**Published:** 2024-09-12

**Authors:** Nilay Aksoy, Nur Ozturk, Tamas Agh, Przemyslaw Kardas

**Affiliations:** ^1^Department of Clinical Pharmacy, Faculty of Pharmacy, Altınbaş University, Istanbul, Türkiye; ^2^Graduate School of Health Sciences, Clinical Pharmacy PhD Program, Istanbul Medipol University, Istanbul, Türkiye; ^3^Syreon Research Institute, Budapest, Hungary; ^4^Medication Adherence Research Group, Center for Health Technology Assessment and Pharmacoeconomic Research, University of Pécs, Pécs, Hungary; ^5^Medication Adherence Research Centre, Department of Family Medicine, Medical University of Lodz, Lodz, Poland

**Keywords:** antirheumatic drugs, medication adherence, prescription claims, medication event monitoring system, patients, meta-analysis

## Abstract

**Introduction:**

This systematic review and meta-analysis aimed to analyze the adherence rate for conventional and biological disease-modifying antirheumatic drugs (DMARDs) utilizing different assessment measures.

**Method:**

A systematic literature search was performed in four electronic databases, including PubMed, Scopus, Web of Science, and the Cochrane Central Register of Controlled Trials (CENTRAL), covering the time frame from April 1970 to April 2023. Studies that present data on medication adherence among adult patients with rheumatoid arthritis (RA), specifically focusing on DMARDs (conventional or biological), were included in the analysis. The adherence rate for different assessment measures was documented and compared, as well as for conventional and biological DMARDs. A random-effects meta-analysis was performed to assess adherence rates across different adherence assessment measures and drug groups.

**Results:**

The search identified 8,480 studies, out of which 66 were finally included in the analysis. The studies included in this meta-analysis had adherence rates ranging from 12 to 98.6%. Adherence rates varied across several adherent measures and calculation methods. Using the subjective assessment measures yielded the outcomes in terms of adherence rate: 64.0% [0.524, 95% CI 0.374–0.675] for interviews and 60.0% [0.611, 95% CI 0.465–0.758] for self-reported measures (e.g., compliance questionnaires on rheumatology CQR-5), *p* > 0.05. In contrast, the objective measurements indicated a lower adherence rate of 54.4% when using the medication event monitoring system (*p* > 0.05). The recorded rate of adherence to biological DMARDs was 45.3% [0.573, 95% CI 0.516–0.631], whereas the adherence rate for conventional DMARDs was 51.5% [0.632, 95% CI 0.537–0.727], *p* > 0.05. In the meta-regression analysis, the covariate “Country of origin” shows a statistically significant (*p* = 0.003) negative effect with a point estimate of −0.36, SE (0.12), 95% CI, −0.61 to −0.12.

**Discussion:**

Despite its seemingly insignificant factors that affect the adherence rate, this meta-analysis reveals variation in adherence rate within the types of studies conducted, the methodology used to measure adherence, and for different antirheumatic drugs. Further research is needed to validate the findings of this meta-analysis before applying them to clinical practice and scientific research. In order to secure high reliability of adherence studies, compliance with available reporting guidelines for medication adherence research is more than advisable.

## 1 Introduction

Rheumatoid arthritis (RA) is a chronic autoimmune disease that is characterized by persistent inflammation of the synovial membrane (synovitis), systemic inflammation, and autoantibodies (1). The tendon sheaths and bursae synovia are also affected by the inflammation. Furthermore, the presence of inflammatory substances such as interleukin-1(IL-1), IL-17, and nitrogen intermediates leads to a depletion of chondrocytes in cartilage, ultimately resulting in apoptosis and cartilage degradation (2). RA impacts more than 20 million individuals globally, exhibiting a greater prevalence among females and the geriatric population (3). The annual incidence of RA in European countries ranges from 20 to 50 cases per 100,000 individuals (4, 5). Furthermore, in 2020, the age-standardized global prevalence rate of RA was 208.8 cases per 100,000 individuals. The prevalence was higher in females, with a rate of 293.5 per 100,000 individuals, compared to males with a rate of 119.8 per 100,000 individuals (6).

RA significantly impacts patient’s quality of life. It is characterized by persistent discomfort, stiffness in the joints, and fatigue, all of which hinder physical activity and mobility, resulting in a person’s dependence on others. Moreover, RA can cause prolonged psychological distress since individuals may get disappointed with the ongoing challenges of managing a chronic illness. Uncontrolled RA has been found to result in joint deterioration, disability, reduced quality of life, and the development of cardiovascular diseases and other comorbidities (7).

A variety of pharmacological and non-pharmacological interventions are employed in the management of autoimmune rheumatic conditions. Pharmacological treatments such as corticosteroids, nonsteroidal anti-inflammatory drugs (NSAIDs), analgesics, and disease-modifying antirheumatic drugs (DMARDs) are among the numerous options available. The two primary categories of DMARDs are biological DMARDs (bDMARDs) and nonbiological DMARDs which include conventional synthetic DMARDs (cDMARDs) and targeted synthetic DMARDs (tsDMARDs) (8). Although cDMARDs have many advantages, such as low cost, widespread availability, long-term usage, and the flexibility to combine them, they also have some drawbacks, such as widespread immunosuppression, delayed onset of action, and the need for frequent monitoring. Targeted therapy, rapid onset of action, and efficacy for non-responders are all advantages of bDMARDs and tsDMARDs that helped overcome cDMARDs’ drawbacks. However, the agents’ high cost, immunogenicity, and infection risk prevent their widespread usage (9). The clinical practice guideline functions as a tool to assist clinicians and patients in making well-informed decisions regarding the most appropriate medication for the patient, taking into account all relevant factors (10).

Medication adherence refers to the act of individuals following the prescribed regimen for medication consumption with precision. Medication adherence is described by its three major components: (a) initiation, which occurs when a patient takes the first dose of prescribed medication; (b) execution adherence, which occurs when a patient’s actual dosing corresponds to the prescribed dosing regimen from initiation until the last dose is taken; and (c) persistence, which occurs when a patient fills prescription without gaps (11).

The medical literature has examined various factors that may contribute to non-adherence to RA medications. These factors include, side effects, did not experience a benefit from the drug (12–14), the complexity of drug regimens (8, 15), the cost of medication (16, 17), inadequate information and patient education, psychological factors, cognitive impairments, logistical challenges, beliefs and attitudes, stigma and social support, patient-related factors such as age, health literacy, education level, and perceived ineffectiveness (18, 19). In addition, disease severity and clinical characteristics of RA can influence adherence rate for instance, patients with longer duration of disease had poor mental health and higher disease activity had shown lower adherence rate compared to the patients with shorter duration (20). Likewise, medication adherence rate might also be influenced by belief of patients about medicines and diseases (21). The prevalence of non-adherence to RA medications is widely based on these factors; many studies reported adherence rates to antirheumatic drugs ranging between 30 and 80% (22). It is crucial to acknowledge that a significant challenge in interpreting the results of the studies of adherence lies in the heterogeneity of the definition and measures used. This is true despite the availability of relevant terminology frameworks (23) and reporting guidelines (24), which may contribute to the variations observed in adherence levels to RA medications.

The importance of involving patients in the decision-making process underscores the necessity of investigating the concept of adherence in the context of chronic illnesses. Failure to comply with RA therapy may lead to treatment failure, delayed recovery, accelerated disease progression, and necessitate more aggressive treatment. Furthermore, patients with RA typically have concomitant comorbidities and are therefore equipped with polypharmacy, which further exacerbates the challenges associated with medication adherence (25).

The full advantages of DMARDs can be obtained by patients who carefully adhere to their medication regimens. However, despite being the primary treatment for inflammatory rheumatic diseases, DMARDs often suffer from low adherence rates (26). As per prior research, non-adherence to DMARDs has been found to be associated with heightened disease activity, functional impairment, and reduced quality of life (27). Therefore, the primary objective of this study was to analyze the adherence rate for DMARDs as well as capture the diversity in adherence rates across different measures that use different calculation methods and between patients taking cDMARDs and bDMARDs. This objective was addressed by conducting a systematic literature review (SLR) and meta-analysis, with the goal of offering a thorough and quantitative summary of the available evidence.

## 2 Methods

This study presents a systematic review and meta-analysis in accordance with the revised Preferred Reporting Items for Systematic Reviews and Meta-Analyses (PRISMA) statement (28) and study was performed according to the PIO (Population: patients with RA, I: DMARDs as intervention, O: adherence rate as outcomes) while the research question of the study was: What are the adherence rates for DMARDs as well as capture the diversity in adherence rates across various studies assessing RA therapy?

### 2.1 Literature search strategy

We performed a systematic search of four electronic databases: PubMed, Scopus, Web of Science, and the Cochrane Central Register of Controlled Trials (CENTRAL) from April 1970 until April 2023. We used a combination of the following keywords to build the search strategy: (Arthritis, Rheumatoid OR RA) AND (Adherence OR compliance OR nonadherence OR non-adherence OR noncompliance OR non-compliance OR continuation OR persistence OR concordance OR “continuation rates” OR “continuation rate”). Detailed search strategies for different databases are mentioned in Supplementary Table S1.

### 2.2 Inclusion and exclusion criteria

To address our study’s aim, our study included patients diagnosed with RA who were ≥ 18 years of age. The search focused on articles that provided data on adherence to antirheumatic drugs. Additionally, we specifically sought studies that documented the method used to determine adherence. Furthermore, cross-sectional, prospective, retrospective, observational studies and randomized controlled trials (RCTs) published in English language during April 1970 to April 2023 were included.

The exclusion criteria encompass studies about rheumatology diseases other than RA, adherence to non-pharmacological therapies, articles that solely discuss persistence, discontinuation, switching, or retention rates without providing information on adherence, articles lacking precise methods or cutoff points for measuring adherence, reviews, case series, case reports, commentaries, letters to editors, articles published before April 1970, and articles published in language other than English.

**Figure 1:**
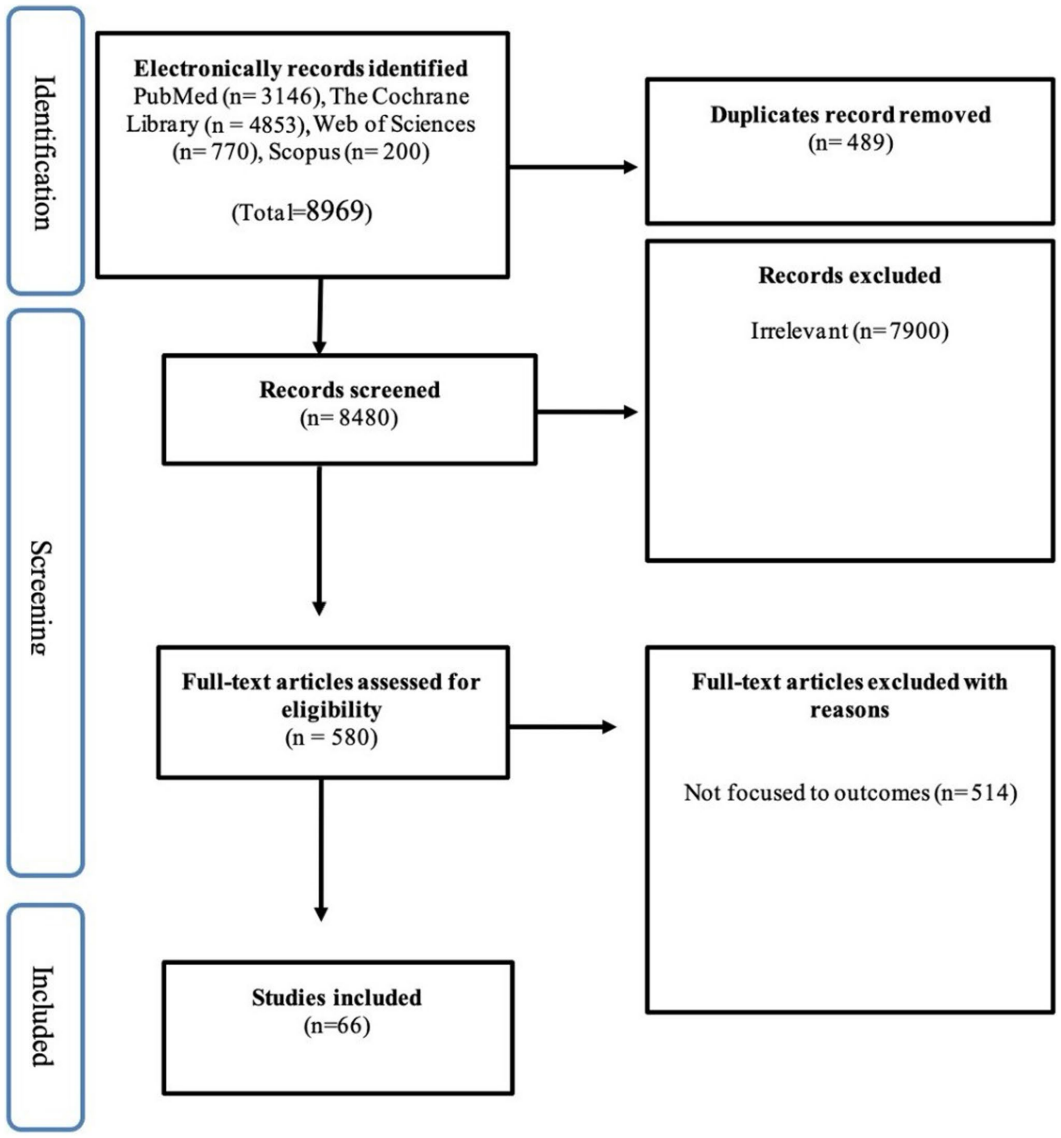
PRISMA flow chart.

A thorough and exhaustive search was undertaken on the articles included in the review, duplicate studies were carefully evaluated and then excluded. Afterwards, two authors, NA and NO, conducted separate assessments of all abstracts and titles using Endnote to find out their appropriateness for inclusion. After conducting an initial screening of titles and abstracts, publications that satisfied the eligibility requirements were subjected to a comprehensive examination and evaluation by two authors separately to verify their suitability for inclusion based on the predetermined criteria. When disparities emerged and a unanimous agreement could not be attained, a third author (PK) was consulted to render a conclusive decision. Figure 1 depicts the diagram of the study flow.

### 2.3 Study selection and data extraction

The data obtained from the studies that met the inclusion criteria was consolidated into an MS Excel spreadsheet. This dataset encompasses various variables such as the gender and age distribution of the study population, study design, country of origin, population size, the time point at which adherence was assessed, prevalence of adherence, type of disease-modifying agents utilized, and the specific measurement employed to determine the adherence rates. The data retrieved from RCTs were exclusively for the control group, aiming to mimic the data from the other studies that were included.

### 2.4 Quality assessment

We assessed the quality of the included RCTs according to the Cochrane Handbook of Systematic Reviews of Interventions using the Risk of Bias Tool, which admits the following six domains: random sequence generation, allocation concealment, blinding of participants and personnel, blinding of outcome assessment, incomplete outcome data, selective outcome reporting, and other potential sources of bias. Domains were evaluated by the authors, who assigned them a bias risk level of “low,” “high,” or “unclear” (29).

The New Castle Ottawa scale was utilized to evaluate cross-sectional, prospective, retrospective and observational studies. This scale encompasses three primary domains, namely selection, comparability, and outcome domains (30).

### 2.5 Data synthesis

The data analysis was conducted using Open Meta [analyst] Software (version 12.11.14) for making the forest plots with labels and Microsoft Excel for the forest plots without labels. To account for observed heterogeneity in outcomes, a random effect model was utilized to pool continuous data as mean and standard deviation with a 95% confidence interval (CI). The heterogeneity was measured by the Cochrane Q test and I-square statistic, and the results were significantly heterogeneous when *p* < 0.05 and I2 ≥ 50% (31). The levels of heterogeneity were determined according to the Cochrane Handbook as low if *I*^2^ = 25%, moderate if *I*^2^ = 50%, and high if *I*^2^ = 75%. The results were statistically significant if the *p* value was <0.05. A meta-regression study was performed to examine the impact of drug type and various assessment methods on the adherence rate.

## 3 Results

### 3.1 Literature searched

Different databases, including PubMed, The Cochrane Library, Web of Sciences, and Scopus were searched for the relevant literature. Initially, 8,969 research articles were retrieved. In the identification phase of PRISMA, 489 research papers were found to be duplicated and removed before starting the titles and abstracts screening utilizing Endnote X9. During the screening phase, 8,480 research papers were evaluated for their eligibility. After a thorough screening and following the eligibility criteria, 7,900 research papers were excluded. After screening, only 580 research papers were found eligible for full-text assessment. In the last phase, only 66 research papers were included, as indicated in Figure 1.

### 3.2 Eligible studies

Among the 66 studies that were included in the analysis, 22 were cross-sectional in nature, 21 were prospective, 20 were retrospective, and only 3 were RCTs. The cumulative sample size of the studies under consideration is 136,619 individuals diagnosed with RA. The specific characteristics of the included studies are summarized in Table 1.

**Table 1:** The general characteristics of the included studies.

Author and year	Study design	Country	Population number	Duration of follow up (months)	Medications	Age (years)		Females
						Mean	SD	%
Lee and Tan (76)	Cross-sectional	New Zealand	108	NA	Antirheumatic tablets	54.5	13.9	78.7
Owen et al. (77)	Cross-sectional	Australia	178	NA	NSAID, Corticosteroids, slow-acting antirheumatic drugs	NA	NA	69.7
Pullar et al. (63)	Cross-sectional	United Kingdom	26	NA	D-Pen	54.2	13.5	NA
Lorish et al. (78)	Cross-sectional	United States	200	NA	Arthritis medications	51	27	58.0
Brus et al. (64)	RCT	Netherlands	30	6	Sulfasalazine	58.7	9.2	70.0
Park et al. (62)	Prospective	United States	121	1	Arthritis medications	56	12.7	82.6
Hill et al. (58)	RCT prospective	United Kingdom	49	6	D-Pen	59.2	13	79.6
Harley et al. (38)	Retrospective	United States	2,662	NA	Methotrexate, entracept, Infliximab	51.5	14.2	73.3
Tuncay et al. (79)	Prospective	Turkey	86	12	NSAID, Corticosteroids, DMARD	49.3	11.8	84.9
Borah et al. (80)	Retrospective	United States	3,829	NA	Adalimumab, etanercept	49.6	12.9	77.1
van den BEMT et al. (14)	Cross-sectional	Netherlands	228	NA	DMARD	56.2	12.2	67.5
Contreras-Ya’n˜ez et al. (81)	Prospective	Mexico	93	6	DMARD	40.8	13.9	93.0
Li et al. (48)	Retrospective	United States	2,638	NA	Anakinra, entracept, Infliximab	58.2	15.8	81.0
Salt and Frazier (82).	Cross-sectional	United States	108	NA	Oral DMARD, biologics, staeroids	52	13	75.9
CANNON et al. (83)	Retrospective	United States	455	NA	Methotrexate	64	11	7.9
van den BEMT et al. (84)	Prospective	Netherlands	50	NA	Oral DMARD	55.2	12.4	70.0
Jinnett and parry (37).	Retrospective	United States	447	NA	Oral DMARD, Biologics. Not specified.	52.3	9.5	61.7
Waimann et al. (20)	Prospective	United States	107	24	Methotrexate, Leflunomide, Hydroxychloroquine, Sulfasalazine, Prednisone	NA	NA	86.9
Degli Esposti et al. (36)	Retrospective	Italy	438	36	Adalimumab, tanercept, infliximab	49.6	14.6	53.1
Tkacz et al. (85)	Retrospective	United States	3,892	NA	Adalimumab, Entracept, golimumab	51.1	11.2	75.5
Treharne et al. (32)	Cross-sectional	United Kingdom	85	NA	DMARD, NSAID, steroid	58.8	12.6	75.3
Bluett et al. (41)	Prospective	United Kingdom	392	6	Adalimumab, etanercept, certolizumab, golimumab	57.6	4.1	74.5
Forsblad-d’Elia et al. (42)	Prospective	Sweden	530	24	Tocilizumab	57.8	12.7	80.6
Pasma et al. (49)	Prospective	Netherlands	120	12	Methotrexate and DMARDs	55.7	13.2	66.7
Chu et al. (39)	Retrospective	United States	2,151	NA	Adalimumab, etanercept	NA	NA	81.4
Jørgensen et al. (86)	Prospective	Denmark	772	24	Adalimumab, etanercept, and tocilizumab	56	12.9	76.9
Sharma et al. (87)	Cross-sectional	India	100	NA	Anti-rheumatic drugs	NA	NA	100
Arshad et al. (88)	Cross-sectional	Pakistan	100	NA	Methotrexate	41.5	11.2	73.0
De Cuyper et al. (45)	Prospective	Belgium	129	4	Methotrexate	61	NA	59.7
Prudente et al. (35)	Cross-sectional	Brazil	55	NA	Anti-rheumatic drugs	NA	NA	92.4
Müller et al., 2017 (89)	Retrospective	Germany	7,146	NA	Methotrexate	64.4	12.7	73.6
Calvo-Alén et al. (90)	Cross-sectional	Spain	363	NA	Biologic DMARDs	54.9	12.5	77.7
Gendelman et al. (91)	Prospective	Israel	292	12	Adalimumab	53	14.4	81.2
Calip et al. (57)	Retrospective	United States	53,477	NA	Adalimumab, etanercept, certolizumab pegol or golimumab	NA	NA	67.0
Lathia et al. (92)	Prospective	Canada	4,666	84	DMARDs	69.9	5.46	75.0
Marras et al. (93)	Cross-sectional	Spain	271	NA	Biologics	55.6	12	76.8
Mena-Vazquez et al. (94)	Cross-sectional	Germany	178	NA	DMARDs	56.9	11.7	77.5
Wabe et al. (47)	Prospective	Australia	111	NA	DMARDs	57.9	4.6	57.7
Zhang et al. (95)	Cross-sectional	China	70	NA	Anti-rheumatic drugs	NA	NA	NA
Nakagawa et al. (96)	Prospective	Japan	475	12	Methotrexate, DMARD, prednisolone and biologics	NA	NA	80.8
Suh et al. (97)	Cross-sectional	Korea	292	NA	Methotrexate and biologics	59.3	NA	82.2
Stolshek et al. (98)	Retrospective	United States	10,374	NA	Abatacept, Adalimumab, Certolizumab pegol, Etanercept, Golimumab, Infliximab	49.6	9.7	76.1
Xia et al. (27)	Cross-sectional	China	122	NA	DMARDs	55.2	11.08	85.2
Vogelzang et al. (59)	Prospective	Netherlands	292	36	Etanercept	53.2	5.48	81.8
López-Medina et al. (26)	Cross-sectional	France	1,000	NA	Methotrexate and bDMARDS	NA	NA	80.6
Heidari et al. (33)	Cross-sectional	Iran	308	NA	Antirheumatic tablets	NA	NA	86.0
Salaffi et al. (43)	Prospective	Italy	206	12	Anti TNF	56.9	11.1	68.4
Wabe et al. (99)	Prospective	Australia	110	12	Methotrexate, hydroxychloroquine and sulfasalazine	60	5.3	65.5
Berner et al. (100)	Cross-sectional	Australia	120	NA	Corticosteroids and DMARDs	54	NA	82.5
Oh et al. (46)	Prospective	Korea	2,694	36	Methotrexate, non-steroidal anti-inflammatory drugs (NSAIDs), glucocorticoids, and biologics	NA	NA	86.3
Khilfeh et al. (40)	Retrospective	Multicenter	456	NA	DMARDs	50	NA	77.9
Kuipers et al. (34)	Cross-sectional	Germany	708	NA	DMARDs	59.5	12.1	72.6
Monchablon et al. (101)	Cross-sectional	France	183	NA	DMARD and Biologics	59	13	73.8
Hope et al. (102)	Prospective	United Kingdom	606	6	Methotrexate	60	13	69.1
Mahran et al. (103)	Observational	Egypt	73	NA	Anti-rheumatic drugs	NA	NA	93.2
Berger et al. (104)	Retrospective cohort	United States	675	NA	Biologic DMARDs	NA	NA	77.0
Ometto et al. (105)	Cross-sectional	Italy	191	NA	DMARDs	56.2	5.48	74.3
Pombo-Suarez et al. (8)	Cross-sectional	Spain	859	NA	Corticosteroids and DMARDs	60.2	12.6	77.8
Peter et al. (106)	Retrospective	United States	772	NA	DMARDs	55	13	78.6
Hartman et al. (61)	RCT prospective	Multicenter	419	NA	DMARDs	73	5	69.5
Ubaka et al. (107)	Cross-sectional	Nigeria	169	NA	Anti-rheumatic drugs	NA	NA	62.7
Zuckerman et al. (108)	retrospective	United States	3,530	NA	Biologic DMARDs	53.7	6.05	75
Katchamart et al. (109)	Prospective	Thailand	443	NA	Nonsteroidal anti-inflammatory drugs, glucocorticoids, and DMARDs	60.5	11.6	86.7
Santos-Moreno et al. (44)	Prospective	Colombia	173	24	Adalimumab, etanercept, and golimumab	62	9.9	84.0
Yajima et al. (110)	Cross-sectional	Japan	165	NA	Methotrexate	63.5	5.19	86.1
Kang et al. (50)	Prospective	Korea	367	NA	Anti-rheumatic drugs	60	12	72.6

*Not Announced. NSAID, Non-steroidal anti-inflammatory drugs. RCT, prospective: prospective randomized controlled trial. DMARD, Disease-modifying antirheumatic drugs.

The age of the population varied between 40 and 73 years. Most of the included populations were female, with a percentage of 71.42% of all the participants. A summary of the general characteristics of the included studies is presented in Table 1.

### 3.3 Quality assessment

**Figure 2:**
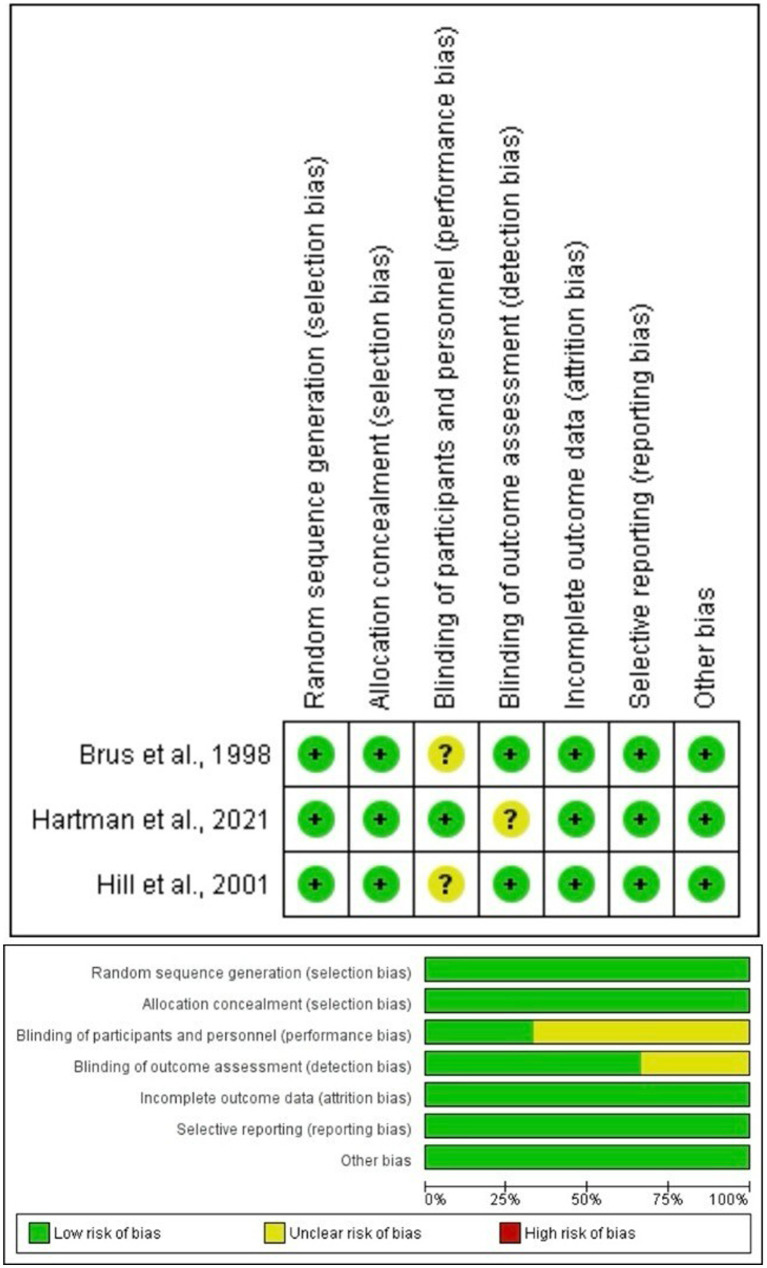
Risk of bias for RCTs.

The 3 included RCTs were of fair quality, showing a low risk of bias in all domains but an unclear risk in the blinding of participants and personnel as there is insufficient information to permit judgment (Figure 2). The fair quality may be due to the dependence of the studies on outcome assessment and the ascertainment of exposure on self-reports.

Most of the included cross-sectional studies showed good quality according to the Newcastle Ottawa scale. Treharne et al. (32), Heidari et al. (33), Kuipers et al. (34), and Prudente et al. (35) showed fair quality because the assessment of outcome and ascertainment of exposure were based on self-reports. Regarding the retrospective studies; Degli Esposti et al. (36), Jinnett and parry (37), Harley et al. (38), Chu et al. (39), and Khilfeh et al. (40) showed fair quality, while all the other studies showed good quality. Nine of the prospective studies showed fair quality, while the other studies are of good quality. These nine studies are Waimann et al. (20), Bluett et al. (41), Forsblad-d’Elia et al. (42), Salaffi et al. (43), Santos-Moreno et al. (44), De Cuyper et al. (45), Oh et al. (46), and Wabe et al. (47) (Table 2).

**Table 2:** Methodological quality assessment for non-RCTs (observational, cross-sectional, retrospective, prospective studies) using New Castle Ottawa scale.

Author and year	Study design	Selection				Comparability	Outcome	Statistics
		Representativeness of the sample	Sample size justified	Non-respondents	Ascertainment of exposure (max**)	Confounding controlled (max**)	Outcome assessment (max**)	
Lee and Tan (76)	Cross-sectional	*	*	*	**	**	**	*
Owen et al. (77)	Cross-sectional	*	*	*	**	**	**	*
Pullar et al. (63)	Cross-sectional	*	*	*	**	**	**	*
Lorish et al. (78)	Cross-sectional	*	*	*	**	**	**	*
Park et al. (62)	Prospective	*	*	*	**	**	**	*
Harley et al. (38)	Retrospective	*	*	*	*	**	**	*
Tuncay et al. (79)	Prospective	*	*	*	**	**	**	*
Borah et al. (80)	Retrospective	*	*	*	**	**	**	*
Van den BEMT et al. (14)	Cross-sectional	*	*	*	**	**	**	*
Contreras-Ya’n˜ez et al. (81)	Prospective	*	*	*	**	**	**	*
Li et al. (48)	Retrospective	*	*	*	**	**	**	*
Salt and Frazier (82).	Cross-sectional	*	*	*	**	**	**	*
CANNON et al. (83)	Retrospective	*	*	*	**	**	**	*
van den BEMT et al. (84)	Prospective	*	*	*	**	**	**	*
Jinnett and parry (37)	Retrospective	*	*	*	*	**	**	*
Waimann et al. (37)	Prospective	*	*	*	**	*	**	*
Degli Esposti et al. (36)	Retrospective	*	*	*	*	**	**	*
Tkacz et al. (85)	Retrospective	*	*	*	**	**	**	*
Treharne et al. (32)	Cross-sectional	*	*	*	*	**	**	*
Bluett et al. (41)	Prospective	*	*	*	**	*	**	*
Forsblad-d’Elia et al. (42)	Prospective	*	*	*	**	*	**	*
Pasma et al. (49)	Prospective	*	*	*	**	**	**	*
Chu et al. (39)	Retrospective	*	*	*	*	**	**	*
Jørgensen et al. (86)	Prospective	*	*	*	**	**	**	*
Sharma et al. (87)	Cross-sectional	*	*	*	**	**	**	*
Arshad et al. (88)	Cross-sectional	*	*	*	**	**	**	*
De Cuyper et al. (45)	Prospective	*	*	*	**	*	**	*
Prudente et al. (35)	Cross-sectional	*	*	*	*	**	**	*
Müller et al. (89)	Retrospective	*	*	*	**	**	**	*
Calvo-Alén et al. (90)	Cross-sectional	*	*	*	**	**	**	*
Gendelman et al. (91)	Prospective	*	*	*	**	**	**	*
Calip et al. (57)	Retrospective	*	*	*	**	**	**	*
Lathia et al. (92)	Prospective	*	*	*	**	**	**	*
Marras et al. (93)	Cross-sectional	*	*	*	**	**	**	*
Mena-Vazquez et al. (94)	Cross-sectional	*	*	*	**	**	**	*
Wabe et al. (47)	Prospective	*	*	*	**	*	**	*
Zhang et al. (95)	Cross-sectional	*	*	*	**	**	**	*
Nakagawa et al. (96)	Prospective	*	*	*	**	**	**	*
Suh et al. (97)	Cross-sectional	*	*	*	**	**	**	*
Stolshek et al. (98)	Retrospective	*	*	*	**	**	**	*
Xia et al. (27)	Cross-sectional	*	*	*	**	**	**	*
Vogelzang et al. (59)	Prospective	*	*	*	**	**	**	*
López-Medina et al. (26)	Cross-sectional	*	*	*	**	**	**	*
Heidari et al. (33)	Cross-sectional	*	*	*	*	**	**	*
Salaffi et al. (43)	Prospective	*	*	*	**	*	**	*
Wabe et al. (99)	Prospective	*	*	*	**	**	**	*
Berner et al. (100)	Cross-sectional	*	*	*	**	**	**	*
Oh et al. (46)	Prospective	*	*	*	**	*	**	*
Khilfeh et al. (40)	Retrospective	*	*	*	*	**	**	*
Kuipers et al. (34)	Cross-sectional	*	*	*	*	**	**	*
Monchablon et al. (101)	Cross-sectional	*	*	*	**	**	**	*
Hope et al. (102)	Prospective	*	*	*	**	**	**	*
Mahran et al. (103)	Observational	*	*	*	**	**	**	*
Berger et al. (104)	Retrospective cohort	*	*	*	**	**	**	*
Ometto et al. (105)	Cross-sectional	*	*	*	**	**	**	*
Pombo-Suarez et al. (8)	Cross-sectional	*	*	*	**	**	**	*
Peter et al. (106)	Retrospective	*	*	*	**	**	**	*
Ubaka et al. (107)	Cross-sectional	*	*	*	**	**	**	*
Zuckerman et al. (108)	retrospective	*	*	*	**	**	**	*
Katchamart et al. (109)	Prospective	*	*	*	**	**	**	*
Santos-Moreno et al. (44)	Prospective	*	*	*	**	*	**	*
Yajima et al. (110)	Cross-sectional	*	*	*	**	**	**	*
Kang et al. (50)	Prospective	*	*	*	**	**	**	*

*One point for the quality score. **Two points for the quality score.

### 3.4 Outcome

The number of patients who adhere to DMARDs was recorded in all included studies, and the percentage of adherent patients was computed by dividing that number by the total number of study participants. The studies included in this meta-analysis had adherence rates ranging from 12 to 98.6%. The lowest adherence rate was reported by Li et al. study for bDMARDs (Anakinra Group) and the highest adherence rate was reported by van den Bemt et al. for the cDMARDs (interview group) (14, 48). The forest plot for this outcome is shown in Supplementary Figure S1. A leave-one out test was tried, and heterogeneity was resolved. Subgroup analysis was conducted according to the study type, adherence calculation methods and measures, and the type of medication utilized, whether it was biological or conventional. Regarding the type of study subgroup analysis, cross sectional studies showed an effect estimate of 0.549, 95% CI [0.411–0.687] while the RCT showed an effect estimate of 0.656, 95% CI [0.275, 1.037]. The retrospective and prospective studies showed an effect estimate of 0.602, 95% CI [0.536, 0.667] and 0.604, 95% CI [0.507, 0.701] respectively. The omission of the retrospective studies from the whole study sheet showed an effect estimate of 0.571, 95% CI [0.502–0.640].

Several studies have evaluated various adherence measures and methods of calculation; for example, some have used the medication possession ratio (MPR) and the proportion of days covered (PDC) to calculate adherence, while others have relied on self-reported questionnaires like the validated 5-item or 19 item compliance questionnaires for rheumatology (CQR-5, CQR-19) or interviews as a measure.

**Figure 3:**
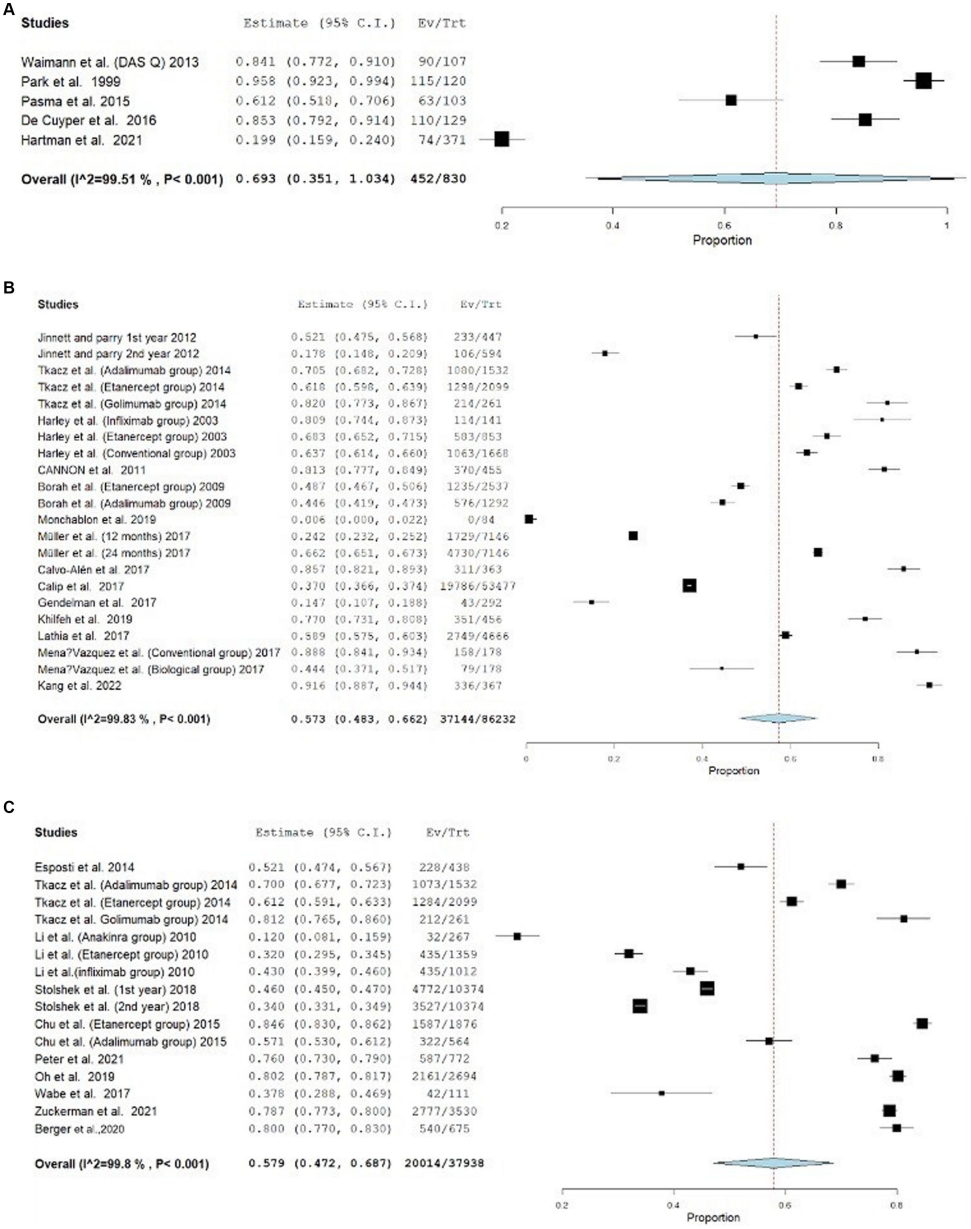
**(A)** Forest plot for adherence outcome in studies assessing adherence by MEMS. **(B)** Forest plot for adherence outcome in studies assessing adherence by MPR. **(C)** Forest plot for adherence outcome in studies assessing adherence by PDC.

**Figure 4:**
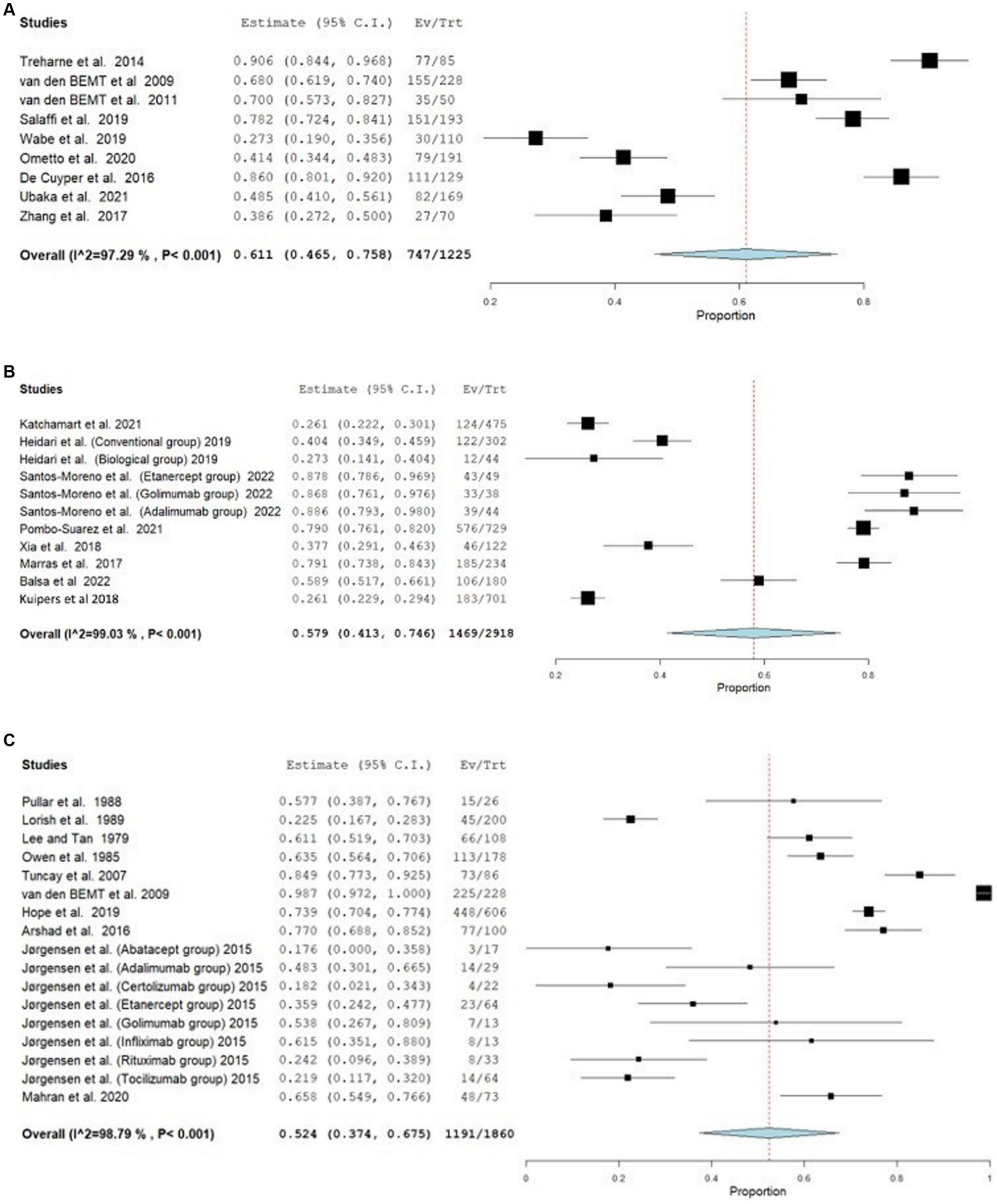
Forest plot of Adherence outcome in studies assessing adherence by CQR-5, CQR-9 and Interviews. **(A)** Forest plot for adherence outcome in studies assessing adherence by CQR-5. **(B)** Forest plot for adherence outcome in studies assessing adherence by CQR-19. **(C)** Forest plot for adherence outcome in studies assessing adherence by interviews.

Furthermore, the effect of the estimates from the studies that employed the medication event monitoring system (MEMS) is 0.693, 95% CI [0.351–1.034], whereas its 0.573, 95% CI (0.483–0.662) and 0.579, 95% CI (0.472–0.687) for MPR and PDC respectively, as shown in figure 3. The effect estimates for the studies that used the CQR-19 and CQR-5 to measure adherence were 0.579, 95% CI [0.413–0.746] and 0.611, 95% CI [0.465–0.758], respectively, while studies that evaluated adherence through interviews had an effect estimate of 0.524, 95% CI [0.374–0.675]. Figure 4 presents the forest plot for these results. The adherence rate was 50, 61, and 64% for CQR-19, CQR-5, and interviews, respectively, *p* > 0.05. The combined comparison of all adherence measures also revealed *p* > 0.05.

**Figure 5:**
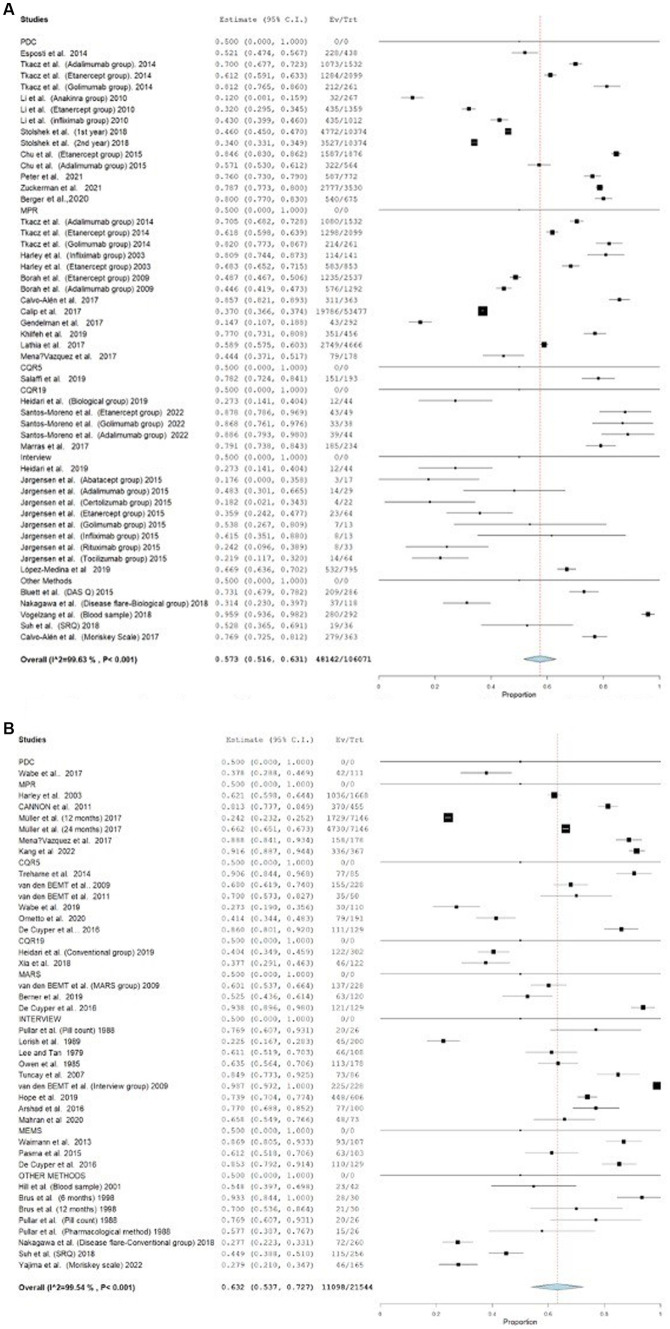
Forest plot for studies assessing adherence to biological and conventional DMARDs. **(A)** Forest plot for studies assessing adherence to biological DMARDs. **(B)** Forest plot for studies assessing adherence to conventional DMARDs.

Additionally, the studies that were included were also examined based on the specific type of the medication. A total of 26 studies examined the patients’ adherence with biological DMARDs. The adherence rates observed in these studies varied from 12 to 95.8%. A total of 29 studies assessed patients’ adherence to conventional DMARDs. The adherence rates for conventional DMARDs varied between 22.5 and 98.6%. The forest plot for these groups is shown in Figure 5. The average adherence rates for biological and conventional DMARDs were 45.15 and 51.5%, respectively (*p* > 0.05).

### 3.5 Meta-regression and correlation

Table 3 presents the results of a meta-regression analysis examining the effect of different covariates on adherence rates. The intercept, with a point estimate of 0.64 and a standard error (SE) of 0.16, is statistically significant (*Z* = 3.90, *p* < 0.001) and indicates the baseline adherence rate when all covariates are at their reference levels. The covariate “Drug type” has a point estimate of 0.11 and an SE of 0.11, with a 95% confidence interval (CI) ranging from −0.11 to 0.34. This suggests that “Drug type” is not a significant predictor of adherence rates (*Z* = 1.01, *p* = 0.31). Similarly, “Measurement type” has a negligible effect on adherence rates, with a point estimate of 0.002 and SE (0.02), and a 95% CI from −0.05 to 0.05. This covariate is also not significant (*Z* = 0.06, *p* = 0.95). Overall, the analysis indicates that the baseline adherence rate is significant, the types of drugs and measurement methods did not significantly influence the adherence rates in this model. The intercept for (origin of studies, quality of studies, study design, and year of the study) has a point estimate of 0.85 with a SE of 0.55, yielding a 95% CI, −0.23 to 1.94, a Z-value of 1.54, and a *p*-value of 0.12. The covariate “Country of origin” shows a statistically significant (*p* = 0.003) negative effect with a point estimate of −0.36, SE (0.12), 95% CI, −0.61 to −0.12. Quality” has a point estimate of −0.13, SE (0.11), 95% CI, −0.36 to 0.08, Z value of −1.20, and *p* value of 0.22, indicating no significant effect. Study design” has a point estimate of 0.03, SE (0.04), 95% CI, −0.05 to 0.12, Z-value of 0.84, and p-value of 0.40, also showing no significant effect. Lastly, “Year” has a negligible point estimate of 0.00 with a 0.36 significance level, indicating no significant effect. The rate of drug adherence for RA therapies across different adherence measures and calculation methods is available in Table 4. Furthermore, the Pearson correlation indicated no correlation between age and adherence rate to RA drugs (r = 0.08, *p* = 0.56).

**Table 3:** Meta-regression outcomes-random effect model.

Covariates	Point estimates	SE	95% CI	*Z* value	*p*- value
Meta-regression for intervention					
Intercept	0.64	0.16	0.32 to 0.96	3.90	<0.001
Drug type	0.11	0.11	−0.11 to 0.34	1.01	0.31
Measurement type	0.002	0.02	−0.05 to 0.05	0.06	0.95
Meta-regression for demographic variables					
Intercept	0.85	0.55	−0.23 to 1.94	1.54	0.12
Country of origin	−0.36	0.12	−0.61 to −0.12	−2.93	0.003
Quality	−0.13	0.11	−0.36 to 0.08	−1.20	0.22
Study design	0.03	0.04	−0.05 to 0.12	0.84	0.40
Year	0.00	0.00	−0.00 to 0.001	0.91	0.36

**Table 4:** Summary of adherence measures and calculation methods.

Adherence measurement methods per data collection method*	No. of studies, *n* (%)	Average adherence rate %	Reference study
MPR for multiple medications: In general, the numerator is the sum of days supplied for a medication and the denominator is the length of the study period. Most studies have at least one variant for either or both the numerator and the denominator			
MPR is defined as the ratio of the duration of DMARD ownership within a specific year (no. days supplied with DMARD) divided by the number of days in the reference year, for those who had at least one DMARD prescription filled in that year.	3 (4.5%)	44.9%	Jinnett and Parry (37), Borah et al. (80), and Müller et al. (89)
MPR is calculated by dividing the total number of days’ supply of the index therapy by the length of treatment.	2 (3%)	68.1%	Tkacz et al. (85) and Cannon et al. (83)
The adherence ratio is calculated by dividing the number of therapeutic administrations or completed prescriptions by the predicted number.	1 (1.5%)	66.1%	Harley et al. (38)
MPR was determined by dividing the total number of drugs delivered to the patient by the total number of prescriptions prescribed over the 6-month period leading up to the day the questionnaire was completed.	2 (3%)	53.9%	Monchablon et al. (101) and Mena-Vazquez et al. (94)
MPR = (number of days actually covered by the medication administered by the patient/ number of days of the study period –theoretically covered by the medication prescribed-) × 100	1 (1.5%)	85.6%	Calvo-Alén et al. (90)
MPR is calculated by dividing the number of days’ supply of medication supplied by the number of days for which the patient was prescribed the medication.	1 (1.5%)	37%	Calip et al. (57)
The (MPR) is a measure that calculates the ratio of the number of days on which medication was distributed to the total number of days from the first dispensation to the final supply day of the last dispensation in the follow-up period, or until cessation of 180 days or more.	2 (3%)	56.3%	Gendelman et al. (91) and Lathia et al. (92)
MPR was determined by dividing the total number of days’ supply of medication by the total number of days of eligibility, with a maximum value of 1.0.	1 (1.5%)	77%	Khilfeh et al. (40)
Adherence was determined based on the medication possession ratio observed during the follow-up period. Individuals who adhered to at least 80% of their prescribed MTX dosages	1 (1.5%)	91.3	Kang et al. (50)
PDC: typically, the numerator represents the total number of days on which a medicine was taken, while the denominator represents the duration of the research period. The majority of studies include at least one variation for either the numerator, the denominator, or both.			
PDC = total mg of the drug prescribed/defined daily dose; total coverage (%) = sum of prescription coverage (days)/duration of the followup period (365 days) × 100.	2 (3%)	36.7%	Degli Esposti et al. (36) and Li et al. (48)
The PDC was also computed, taking into account the duplication of covered days.	1 (1.5%)	66%	Tkacz et al. (85)
PDC was determined by dividing the entire number of days a patient’s prescriptions for the index biologic were provided by the total number of days in the corresponding follow-up period.	3 (4.5%)	45.1%	Stolshek et al. (98), Chu et al. (39), and Peter et al. (106)
The adherent group consists of individuals who have not taken their medicine for less than 20 days.	1 (1.5%)	80.2%	Oh et al. (46)
Patients were categorized as adherent if the percentage of days covered for each DMARD was equal to or more than 80%.	2 (3%)	77.4%	Wabe et al. (47) and Zuckerman et al. (108)
PDC was determined by calculating the ratio of the number of days the patient had medicine available during the observation period to the duration of the observation period.	1 (1.5%)	80%	Berger et al. (104)
MEMS			
The evaluation of these vacancies was conducted through patient interviews and a direct comparison with the pharmacy refill record.	1 (1.5%)	84%	Waimann et al. (20)
If a patient consumed the correct dosage on any given day, they were deemed to be adherent.	1 (1.5%)	95.8%	Park et al. (62)
If there was underutilization of medicine, for every patient and every DMARD, every day. The underutilization is registered when the number of actual openings is less than the number of anticipated openings.	1 (1.5%)	61.2%	Pasma et al. (49)
If the patient followed the prescription to the letter and opened the MEMS container at least once in a week, we deemed them totally adherent. Every patient was assigned a score of 1 (opened) or 0 (not opened) for each of the 16 weeks in a row. Medication adherence was calculated as the mean of these 16 assessments and then multiplied by 100.	1 (1.5%)	85.3%	De Cuyper et al. (45)
Using the first pattern seen in 50% or more of the patients, we classified their drug adherence pattern. non-user<20% stable user ≥80% weekly users: one opening per week irregular users: different or unclassifiable.	1 (1.5%)	19.9%	Hartman et al. (61)
Self-reported measures			
CQR-5			
How often they forget to take medications /miss /adjust a dose (5-point scale from very often to never). Adherent patients were defined as rarely or never miss a dose. CQRscore ≥80%	9 (13.6%)	60.9%	Treharne et al. (32), Van den Bemt et al. (14), Van den Bemt et al. (84), Salaffi et al. (43), Wabe et al. (99), Ometto et al. (105), De Cuyper et al. (45), Ubaka et al. (107), and Zhang et al. (95)
CQR-19			
The CQR-19, or adherence Questionnaire for Rheumatology, was used to measure medication non adherence. A perfect score of 100 would indicate full adherence, while a score of 0 would indicate non adherence. CQRscore ≥80%	8 (12.1%)	50.3%	Katchamart et al. (109), Heidari et al. (33), Santos-Moreno et al. (44), Pombo-Suarez et al. (8), Xia et al. (27), Marras et al. (93), Balsa et al. (12), and Kuipers et al. (34)
MARS			
Keep forgetting to take, change the dosage, discontinue taking, skip a dose, etc. Five-point scale; never = 5 to very often =1. MARS-9RA Scores range from 9 to 45. Score > 39 considered adherent.	1 (1.5%)	90.7%	Salt and Frazier (82)
Questions regarding taking medications and missing doses (4-point scale, 0 = strongly disagree, 3 = strongly agree). MARS total score > 23. Score 5–25	1 (1.5%)	60%	Van den Bemt et al. (14)
MARS-5 score of 25. Score 5–25	1 (1.5%)	52.5%	Berner et al. (100)
The MARS-5 score range was 5–25; with respondents having scores ≥15 termed as ‘adherent’	2 (3%)	76.8%	Ubaka et al. (107) and De Cuyper et al. (45)
MMAS-8	2 (3%)	60.6%	Yajima et al. (110) and Monchablon et al. (101)
MMAS-4	2 (3%)	77.5%	Prudente et al. (35), Calvo-Alén et al. (90), and Monchablon et al. (101)
Behavioral self-reported Question (1 question)	1 (1.5%)	73%	Bluett et al. (41)
CQ-adherence locally designed	1 (1.5%)	50.5%	Conteras-Ya n ez et al. (81)
Interview	9 (13.6%)	64%	Pullar et al. (63), Lorish et al. (78), Lee and Tan (76), Owen et al. (77), Tuncay et al. (79), Van den bemt et al. (14), Arshad et al. (88), Jorgensen et al. (86), and Mahran et al. (103)
Pill count	3 (4.5%)	88.2%	Brus et al. (64), Pullar et al. (63), and Hartman et al. (61)
Blood sample	2 (3%)	90.7%	Hill et al. (58) and Vogelzang et al. (59)
Use Pharmacological indicator	1(1.5%)	57.6%	Pullar et al. (63)
Adherence assessed by doctor	1 (1.5%)	89.7%	Kuipers et al. (34)

*It should be noted that some research used more than one method to assess adherence. MPR, Medication Possession Ratio; DMARD, Disease modifying agents; MTX, Methotrexate; PDC, proportion of days covered; MEMS, medication event monitoring system; CQR, Compliance-Questionnaire-Rheumatology; MARS, Medication Adherence Report Scale. Use Pharmacological indicator: Low dose phenobarbitone was added to the penicillamine formulation as a pharmacological indicator of compliance. Adherence assessed by doctor: it’s a composite score consisting of physician rating (1 = very adherent, 0 = less adherent) and the match between physicians’ prescriptions and patients’ accounts of their medications (1 = perfect match, 0 = no perfect match).

## 4 Discussion

This meta-analysis set out to describe and contrast the rate of drug adherence for RA therapies across different adherence measures and calculation methods. The adherence rate for antirheumatic drugs in the evaluated studies showed significant variation, ranging from extremely low to nearly perfect adherence. The wide range in adherence rates between these studies may be the result of differing populations under study: Li et al. (48) focused on Medicaid enrollees, who generally have lower socioeconomic status and perhaps other differences in adherence behavior that distinguish them from the broader population included in the study by van den Bemt et al. Furthermore, the methodologies vary in how adherence was measured: Li et al. used the records of administrative claims; van den Bemt et al. (14) used self-reported measure and interview methods, in which adherence may be overestimated because of social desirability bias. The route of administration and treatment regimen of anakinra, given by daily subcutaneous injection, may also contribute to lower adherence rates relative to other biologics with alternative dosing schedules and cDMARDs.

Although the study’s findings are not statistically significant, they showed that adherence rates varied across different assessment methods, medication types (cDMARDs vs. bDMARDs), and calculation methods. Adherence is commonly considered to be the primary factor influencing treatment results in various therapeutic settings. Multiple studies have demonstrated that there is a strong correlation between low adherence and high disease activity in patients with RA (49, 50). According to further research, patient adherence may fluctuate over the course of the disease, as well as in reaction to treatment changes and other contextual factors such as the healthcare system, timing of therapy, and follow-up processes (51). Patient adherence may exhibit variability throughout the duration of the disease, in response to modifications in treatment, and considering additional contextual elements including the healthcare system, therapy schedule, and follow-up procedures (51).

### 4.1 Adherence to conventional and biological DMARDs

The medical literature on conventional and biological DMARD adherence rates is scarce and inconsistent. Blum et al. found that DMARD adherence rates vary substantially. Biological agents have a 41 to 90% adherence rate, while conventional DMARDs have a 30 to 107% adherence rate (52). In the 2023 study by Rosenberg et al. (53), good adherence to biologic and targeted synthetic DMARDs was seen for almost all drugs. Using the PDC method, the proportion of adherent patients ranged from 63.9 to 67.4% in all lines of therapy. This result means that, generally, the rate of adherence was high, particularly for the injectable drugs in comparison with the orally administered drug, irrespective of the status of treatment experience. The highest rate in proportion to adherent patients is noted in drugs taken once every 4–11 weeks: 73.2% in all lines (53).

Van et al. found that medication class, drug load, immediacy of beneficial effects, and side effects did not predict nonadherence in RA patients (22). However, patients adhere better to biological agents than oral DMARDs, according to other reviews (54). We excluded some articles from the biological and conventional forests because they did not define their antirheumatic drugs. In our analysis the biological agents have 12–95.8% adherence rate while conventional DMARDs have 22.5–98.6%.

The variability in adherence rate can be attributed to variations in the measurement method, as well as variations in the definition and threshold for adherence.

Biological agents had significantly greater adherence rates, as determined by both PDC and blood samples, compared to other metrics in the studies included in our meta-analysis.

Conversely, studies that employed MPR as a method of calculation and subjective adherence measures (e.g., interviews and self-reported questionnaires like the CQR) found that biological agents had a lower adherence rate. The findings indicated that there were variations in the mean adherence rate between biological agents and conventional agents, however, these variations did not reach statistical significance. Therefore, despite this discovery, there is insufficient information to conclusively demonstrate that any of these medications have superior adherence compared to one another. Moreover, the meta regression study revealed that the adherence rate was not influenced by the type of medication, be it biological or conventional. Additional variables may have a greater impact on patient adherence.

### 4.2 Different assessment measures of adherence

There was a statistically insignificant difference in the rates of adherence between studies that employed objective measures like MEMS and those that used subjective measures like the Medication Adherence Rating Scale (MARS), interviews, and CQR.

#### 4.2.1 Objective measures

Objective adherence measures such as MEMS, blood sample analysis, pill count, doctor direct observation, and different calculation methods such as PDC and MPR were utilized in 50% of the studies included in our meta-analysis. Objective measures are generally recommended in adherence research because of their numerous benefits. Their benefits include greater precision and reliability, less susceptibility to social desirability bias, real-time monitoring, quantitative data, and early non-adherence identification. However, MPR and PDC are vulnerable to data omissions and uncertainty-related errors (55).

The diversity of the calculation methods was recognized in our meta-analysis. Studies using different PDC and MPR definitions may have inconsistent adherence rates. Fourteen studies used at least nine MPR definitions, whereas 10 used six PDC definitions. Khalifeh et al. (56) defined MPR as ‘total days’ supply divided by total days of eligibility, with a maximum of 1.0′. The study found a 77% adherence rate (56). In contrast, Calip et al. (57) defined MPR as “the proportion of day’ supply of medication dispensed over the number of days the patient was prescribed drugs” and found a 37% adherence rate (57).

Similarly, studies with varied PDC definitions reported different adherence rates. The study by Oh et al. (46) defined “adherent” as a patient who failed to take medication for <20 days. 80.2% of patients met this criterion. Defining the PDC as “total mg of the drug prescribed or defined daily dose; total coverage (%) = sum of prescription coverage (days)/duration of the follow-up period (365 days) × 100” decreases the average adherence rate to 36.7% (36).

Two prospective studies assessed adherence using blood sample analysis for therapeutic drug monitoring (TDM). This is a reliable way for researchers to verify patient medication use with an average adherence of 90.7%. This measurement method involves an intrusive and expensive assay. Additionally, patient-specific factors may cause variations (55). This may explain the decreased use of this measure to assess antirheumatic drug adherence (58, 59).

MEMS was used in five studies. MEMS can accurately measure medication adherence, and dose, and provide continuous monitoring over time (60). Most of these studies compared MEMS to other adherence measures. Waimann et al. (20) compared the CQR ratings of patients who agreed to electronic monitoring with those who rejected (20). Non-adherence was consistently tracked electronically in the Pasma et al. (49) study and was defined as the proportion of days with a negative difference between expected and observed medicine container openings throughout the 3-month period before disease activity measurement (49). De Cuyper et al. (45) used MEMS for 16 weeks with MARS-5 and CQR adherence surveys (45). Hartman et al. (61) compared MEMS to pill counting (61). The oldest study tested patients in a private doctor’s office and tracked their treatment adherence electronically for 1 month at work and home (62). MEMS provides real-time dosage timing data but does not ensure medicine consumption, which may explain the comparability with other adherence measures (55). Our meta-analysis revealed a varying adherence rate due to the various cut-off values and how they disrupted the MEMS container opening pattern. Pill counting tracks medication units and days administered to determine adherence. Three trials measured antirheumatic medication adherence with pill counting. Hartman et al. (61) found that MEMS adherence is lower than pill count in older rheumatoid arthritis patients. Pill count adherence was higher than pharmacological indicator adherence in another trial (63). Finally, one trial assessed adherence purely using pill counting (64). Pill counting improves accuracy and verifies prescription use (65).

#### 4.2.2 Subjective measures

About 42% of studies in our meta-analysis measured adherence with self-reported questionnaires. Self-reported questionnaires are widely utilized in antirheumatic drug adherence studies. The subjective measures are economical, efficient, convenient, non-intrusive, privacy-conscious, and practical. However, these methods are susceptible to bias due to their dependence on self-reporting from participants, which can be affected by biases related to social desirability, memory, or interpretation. Self-reported questionnaires can also assess medication adherence and subsequent lifestyle improvement (66). A comprehensive review for the methods for measuring multiple medication adherence found that 50% of studies assessed multiple drug adherence using self-reported measures for the previously mentioned benefits (67). The most common self-reported questionnaires used in our meta-analysis were the CQR-5 and CQR-19, used in 9 and 8 studies, respectively. The mean adherence rate for CQR-5 studies was higher than CQR-19 studies. CQR-5 and CQR-19 average adherence rates varied due to differences in questionnaire length, specificity, item content, and population factors. CQR-5 is a five-item questionnaire for general drug adherence, while CQR-19 is a more comprehensive assessment of medical topics (68, 69).

Our meta-analysis demonstrated that differences in questionnaire or scale selection can influence adherence rates, as evidenced by the contradictory findings in the five MARS scale studies.

We noted that few studies in our meta-analysis examined antirheumatic therapy adherence using the Morisky generic adherence instruments (MMAS-4 and MMAS-8). Rheumatologists and healthcare professionals may prefer disease-specific measurements or modified adherence scales to better reflect antirheumatic medication problems. Overall, self-reported measures produced a high adherence rate in our meta-analysis, but we must keep in mind that self-reported evaluations have drawbacks such as social desirability bias, memory limitations, and social and cultural influences on respondents’ answers. These factors may alter data reliability and validity (65, 66).

Nine studies mostly measured adherence via interviews. The causes of drug non-adherence were investigated in several unstructured and semi-structured medical literature interviews. These interviews asked patients or caregivers open-ended or closed-ended questions. The interviews were conducted in person, by phone, or via video (70). However, all interviews in our meta-analysis were conducted face-to-face during the visit. The questionnaires are normally administered in clinical settings in a way that allows all the participants to understand the questions being asked and, therefore, answer them accurately. The questions may be read out to those who cannot read or may not understand what is written, or they may be self-administrative for those who can do it themselves. However, the articles included in this meta-analysis do not explicitly mention the use of self-reporting questions being read out to the participants; they mention standardized interviews with the pharmacy consultant or physicians. These interview studies had an average adherence rate of 64%, which is greater than typical self-reported studies. This conclusion aligns with van den BEMT et al. (14), which showed significantly lower self-reported adherence when a questionnaire was provided than when the patient was directly interviewed by a professional pharmacist (14).

Interviews are a flexible tool that offers in-depth medication adherence information, allowing for individualized interaction and real-time adjustments to probe a variety of unexpected responses. However, they are very subjective and hence are subject to several biases, such as interviewer bias, recall bias, and social desirability bias, which threaten the accuracy of the data. The qualitative nature of the data also calls for expert analysis and may have limited generalizability (55, 71). Hence, using multiple methods and data sources may help understand adherence practices.

### 4.3 Factors affecting adherence to RA drugs

In this metanalysis, there was no correlation between the age and adherence rate. On the other hand, some systematic reviews and meta-analyses have tested for the possible relationship between age and adherence to antirheumatic drugs, with conflicting results. Generally, studies suggest that age may be weakly or not at all correlated with adherence rates to disease-modifying antirheumatic drugs, including both conventional and biological agents (72, 73).

Indeed, this meta-analysis had a lengthy search time. However, the year of the study did not have an impact on the adherence rate. On the other hand, the country from which the study originated significantly affected the adherence rate. Countries vary in several aspects, including disparities in healthcare infrastructure, cultural attitudes and beliefs, socioeconomic conditions such as economic barriers, insurance coverage, variations in health system organizations, and differences in patient education and support. Previous literature has established that all of these factors have a significant impact on the adherence rate for DMARD (74).

Despite the surprising nature of the findings, the variations in adherence rates observed in our meta-analysis cannot be attributed to the type of measurements used. This conclusion is supported by the meta-regression analysis, which indicated that the measurement type does not significantly affect adherence rates. The observed variances may be caused by other factors. The complexity of drug regimens (8, 15), the cost of medication (16, 17), inadequate information and patient education, psychological factors, cognitive impairments, logistical challenges, beliefs and attitudes, stigma and social support, and RA severity and clinical characteristics can also affect adherence rate. Patients with longer disease duration, poor mental health, and higher disease activity had lower adherence rates than those with shorter duration (20). Regrettably, our meta-analysis did not investigate these features due to the unavailability of data in numerous research.

Regardless of evaluation method, medication use, or study type, all included trials had adherence rate ranging from 12 to 98.6%. Specifically, cross-sectional studies had 60.9%, randomized control trials 56.9%, prospective cohort studies 63.9%, and retrospective cohort studies 46% adherence rate. Without retrospective studies, the mean adherence rate across all research rose from 47.2 to 58%. Retrospective studies, despite utilizing reliable data such as drug dispensation records, might be constrained by various factors, including recollection bias, loss of follow-up, selection bias, the absence of objective measurements, and challenges in proving the relationship between exposure and result (75). In contrast, prospective RCTs and well-conducted cross-sectional studies use more stringent methods. Prospective studies collect data in real time, randomized trials assure controlled conditions; and cross-sectional studies provide a snapshot of adherence across time.

### 4.4 Sensitivity analysis

Studies showed considerable heterogeneity (*I*^2^ = 98.78, *p* < 0.001). The substantial variability of the sensitivity test suggests numerous factors affect this study’s outcome. Even slight changes to study type, medication, and assessment procedures can substantially impact adherence. Subgroup analysis supports this conclusion.

Meanwhile, high heterogeneity in a meta-analysis can be caused by various reasons. The populations, interventions, and outcome measures investigated, as well as the study design itself, may differ from one primary study to another, which increases heterogeneity. Moreover, differences in study methodology, such as sample size, data collection methods, and quality of data collection, may also affect variability. Furthermore, there is intrinsic clinical diversity within the population studied, including geographic and temporal differences that may produce heterogeneity. In addition, heterogeneity may be enhanced by methodological inconsistencies, such as variation in statistical methods and outcome definitions.

### 4.5 Impact of the study

This study holds significant relevance for health professionals and interest for patients alike. For healthcare providers, understanding the adherence patterns to these medications is crucial for optimizing treatment outcomes and managing rheumatic conditions effectively. By synthesizing existing evidence, the study provides valuable insights into the assessment methods influencing adherence, allowing healthcare professionals to tailor interventions and support strategies to enhance patient adherence, thereby improving disease management and quality of life. For patients, the findings shed light on the importance of medication adherence in controlling their condition and avoiding potential complications, delegating them to actively participate in their treatment journey. Ultimately, the study’s “so what” lies in its contribution to bridging the gap between evidence-based practice and patient-centered care, fostering better treatment adherence and outcomes in rheumatic diseases.

### 4.6 Limitations

Some study limitations should be mentioned. First, data were gained from studies with a variety of designs and patient populations (e.g., from different countries), resulting in heterogeneity. Other factors, such as RA severity, co-medication used by the patients and local healthcare systems, were not addressed due to data scarcity, which may lead to over interpretation of study results as the primary source of adherence rate variability. Although disease severity and health system data were difficult to collect from the included studies, other patient variables such as age were evaluated and shown to have no correlation with total study adherence rate.

## 5 Conclusion

Suboptimal medication adherence in RA patients is linked to worse treatment outcomes, increased disease activity and radiographical damage of joints, poorer physical performance, increased health services and utilization, and reduced quality of life. The adherence rate for antirheumatic medication exhibited variability between studies due to numerous factors. The country from which the study originated significantly affected the patient adherence rates which could be attributed to differences in healthcare infrastructure, cultural attitudes, socioeconomic conditions, and the organization of healthcare systems. Despite its seemingly insignificant factors that affect the adherence rate, this meta-analysis reveals disparities in adherence rate within the types of studies conducted, the methodology used to measure adherence, and for different antirheumatic drugs. Utilizing a combination of several methodologies and research designs can yield a broader understanding of drug adherence within a specific population. Researchers and healthcare practitioners analyzing adherence rates for antirheumatic medications should be well-versed in the possible sources of variance, the cut-off point for interruption of the used measure, the study population and characteristics, and the strengths and weaknesses of each study design. To secure the high reliability of adherence studies, compliance with available reporting guidelines for medication adherence research is more than advisable.

## Data Availability

The original contributions presented in the study are included in the article/Supplementary material, further inquiries can be directed to the corresponding author.
