# Supplementary figures and images for: Adherence to the antirheumatic drugs: a systematic review and meta-analysis
Source: Front Med (Lausanne). 2024 Sep 12;11:1456251. doi: 10.3389/fmed.2024.1456251 (PMC11424425; doi:10.3389/fmed.2024.1456251)

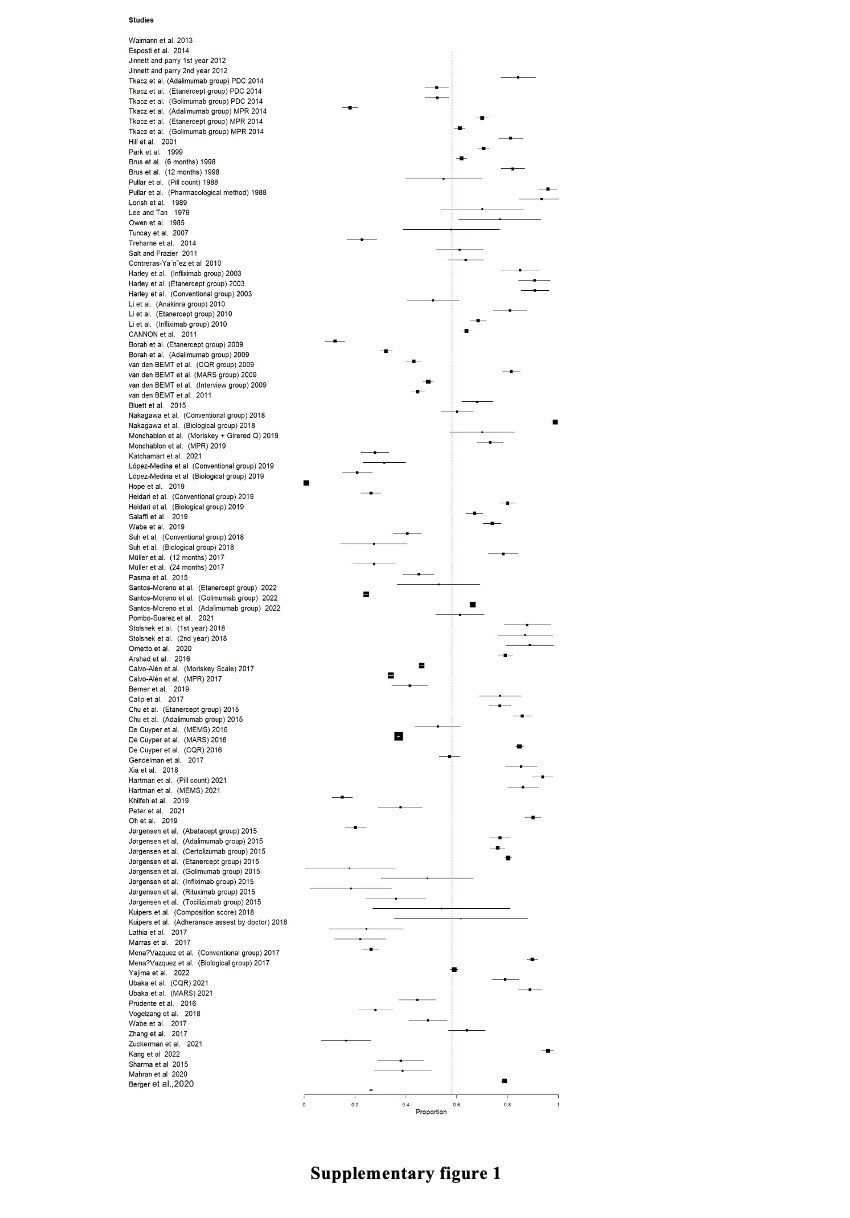

Supplement: Supplementary file 1 [file Image_1.JPEG]
