# Supplementary material for: Adherence to the antirheumatic drugs: a systematic review and meta-analysis
Source: Front Med (Lausanne). 2024 Sep 12;11:1456251. doi: 10.3389/fmed.2024.1456251 (PMC11424425; doi:10.3389/fmed.2024.1456251)
Supplement: Supplementary file 2 [file Table_1.DOCX]

Supplementary table 1.

| Database | Search terms |
| --- | --- |
| PubMed | (("arthritis, rheumatoid"[MeSH Terms] OR "rheumatoid arthritis"[Title/Abstract] OR "RA"[Title/Abstract] OR "rheumatic diseases"[Title/Abstract]) AND ("adherence"[Title/Abstract] OR "compliance"[MeSH Terms] OR "nonadherence"[Title/Abstract] OR "non-adherence"[Title/Abstract] OR "noncompliance"[Title/Abstract] OR "non-compliance"[Title/Abstract] OR "continuation"[Title/Abstract] OR "persistence"[Title/Abstract] OR "concordance"[Title/Abstract] OR "continuation rates"[Title/Abstract] OR "continuation rate"[Title/Abstract])) |
| The Cochrane Library | (“arthritis, rheumatoid” OR “rheumatoid arthritis” OR “RA” OR “rheumatic diseases”)):ti,ab,kw AND ((“adherence” OR “compliance” OR “nonadherence” OR “non-adherence” OR “noncompliance” OR “non-compliance” OR “continuation” OR “persistence” OR “concordance” OR “continuation rates” OR “continuation rate”)):ti,ab,kw |
| Web of Sciences | (“rheumatoid arthritis” OR “RA” OR “rheumatic diseases”) AND (“adherence” OR “compliance” OR “nonadherence” OR “non-adherence” OR “persistence” OR “continuation rates”) |
| Scopus | (“arthritis, rheumatoid” OR “rheumatoid arthritis” OR “RA” OR “rheumatic diseases”) AND (“adherence” OR “compliance” OR “nonadherence” OR “non-adherence” OR “noncompliance” OR “non-compliance” OR “continuation” OR “persistence” OR “concordance” OR “continuation rates” OR “continuation rate”) |
